# Supplementary material for: Quantitation of a Urinary Profile of Biomarkers in Gaucher Disease Type 1 Patients Using Tandem Mass Spectrometry
Source: Diagnostics (Basel). 2022 Jun 8;12(6):1414. doi: 10.3390/diagnostics12061414 (PMC9221757; doi:10.3390/diagnostics12061414)
Supplement: Supplementary file 1 [file diagnostics-12-01414-s001.zip › diagnostics-1715692-supplementary/Supp Figure en PDF/Supplemental Figure S1.pdf]

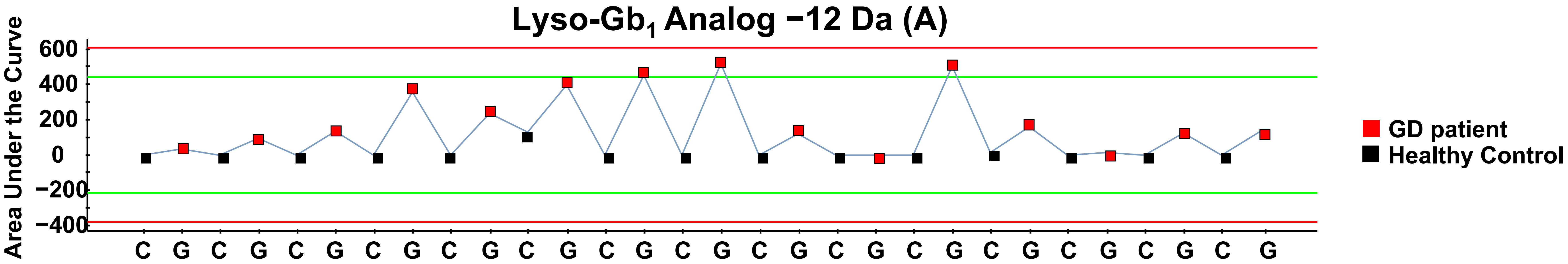

Supplemental Figure S1. Trendplot showing the relative distribution of lyso-Gb_1 analog −12 Da (A) with a retention time of 5.92 min in the different samples analyzed during the metabolomic study. The red square indicates a GD patient (n=15) and a black square indicates a healthy control (n=15). The red line indicates two standard deviations while the green line indicates one standard deviation.
