# Supplementary material for: Quantitation of a Urinary Profile of Biomarkers in Gaucher Disease Type 1 Patients Using Tandem Mass Spectrometry
Source: Diagnostics (Basel). 2022 Jun 8;12(6):1414. doi: 10.3390/diagnostics12061414 (PMC9221757; doi:10.3390/diagnostics12061414)
Supplement: Supplementary file 1 [file diagnostics-12-01414-s001.zip › diagnostics-1715692-supplementary/Supp Figure en PDF/Supplemental Figure S2.pdf]

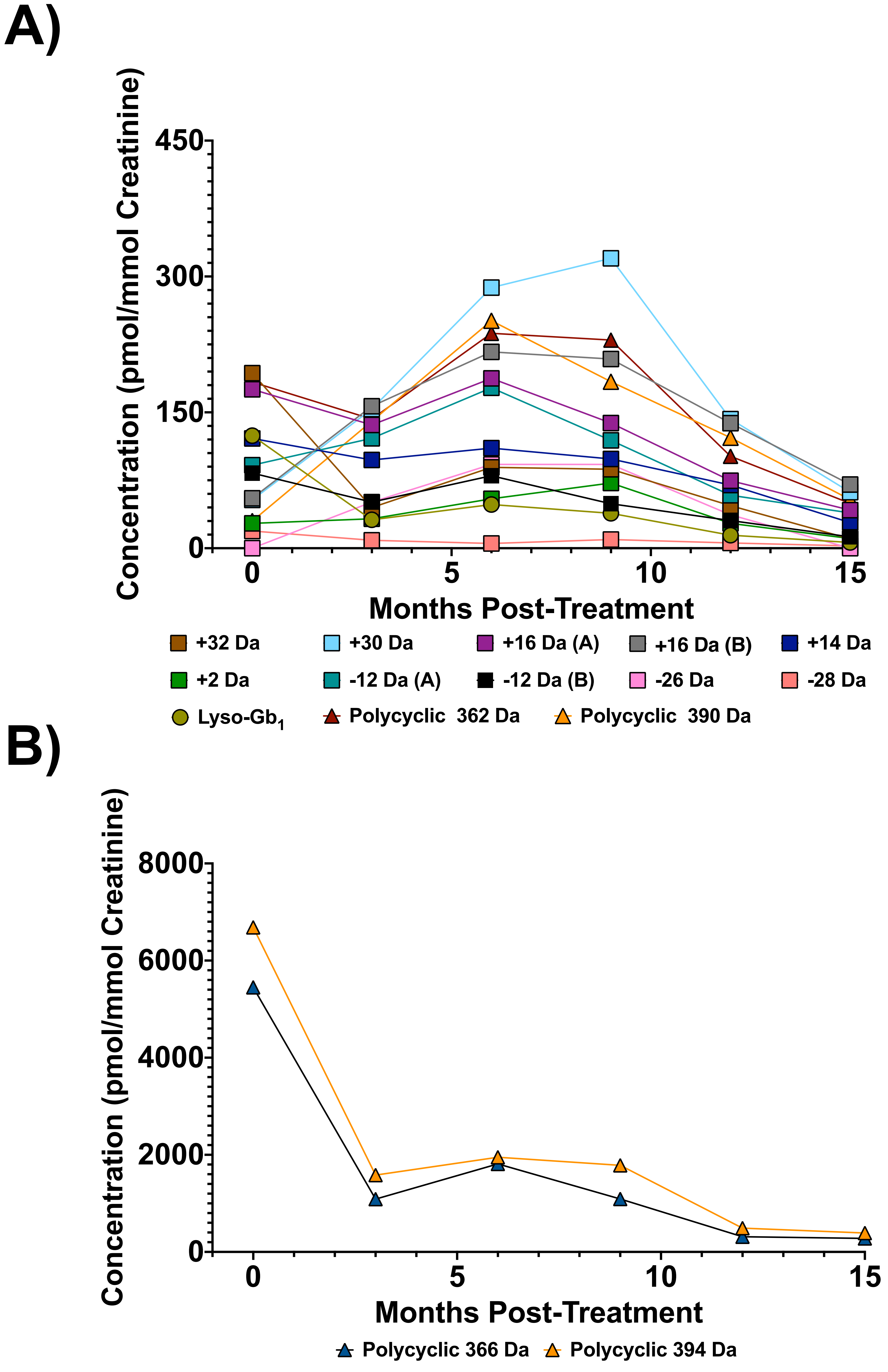

Supplementary Figure S2. Biomarker level variation following ERT treatment in a 55-year-old male GD type 1 patient p.N370S/D409H mutations. A) Concentration variation levels post-ERT for lyso-Gb_1, lyso-Gb_1 analogs +32, +30, +16 (A), +16 (B), +14, +2, − 2 (A) and (B), −26, and −28 Da, as well as polycyclic analogs 362, and 390 Da; B) Concentration variation levels post-ERT for polycyclic lyso-Gb_1 366 and 394 Da.
